# Multifunctional Nano-engineered Polymer Surfaces with Enhanced Mechanical Resistance and Superhydrophobicity

**DOI:** 10.1038/srep43450

**Published:** 2017-03-06

**Authors:** Jaime J. Hernández, Miguel A. Monclús, Iván Navarro-Baena, Felipe Viela, Jon M. Molina-Aldareguia, Isabel Rodríguez

**Affiliations:** 1 Madrid Institute for Advanced Studies in Nanoscience (IMDEA Nanoscience), C/Faraday 9, Ciudad Universitaria de Cantoblanco. 28049 Madrid, Spain; 2 Madrid Institute for Advanced Studies in Materials (IMDEA Materials), C/ Eric Kandel, 2, Tecnogetafe, Getafe. 28906 Madrid, Spain

## Abstract

This paper presents a multifunctional polymer surface that provides superhydrophobicity and self–cleaning functions together with an enhancement in mechanical and electrical performance. These functionalities are produced by nanoimprinting high aspect ratio pillar arrays on polymeric matrix incorporating functional reinforcing elements. Two distinct matrix-filler systems are investigated specifically, Carbon Nanotube reinforced Polystyrene (CNT-PS) and Reduced Graphene Oxide reinforced Polyvinylidene Difluoride (RGO-PVDF). Mechanical characterization of the topographies by quantitative nanoindentation and nanoscratch tests are performed to evidence a considerable increase in stiffness, Young’s modulus and critical failure load with respect to the pristine polymers. The improvement on the mechanical properties is rationalized in terms of effective dispersion and penetration of the fillers into the imprinted structures as determined by confocal Raman and SEM studies. In addition, an increase in the degree of crystallization for the PVDF-RGO imprinted nanocomposite possibly accounts for the larger enhancement observed. Improvement of the mechanical ruggedness of functional textured surfaces with appropriate fillers will enable the implementation of multifunctional nanotextured materials in real applications.

In the last decade a new range of materials has appeared based on structure-mediated surface functionalities produced by surface nanoengineering technologies. A major research undertaken has been in the field of biomimicry^1^. Some of the best known examples are the superhydrophobic surfaces inspired on the lotus leaf or the adhesive surfaces inspired on the gecko feet^2^. These natural surfaces often show multifuntionality. For instance, the gecko feet not only exhibit strong adhesive properties but also possess superhydrophobic and self-cleaning functions^3^. Artificial multifunctional materials are engineered incorporating structural elements and functional elements typically combining enhanced mechanical properties with improved physical (e.g. optical, electrical) and/or chemical (e.g. resistance, biocompatibility) properties^4^.

Multifunctional materials incorporating several functionalities that satisfy multiple uses are attracting a huge interest particularly for the industry to develop new competitive products.

A particular practical approach to engineer surface functions is by nanoimprinting surface textures in polymeric materials. This technique combines texture design and reproduction precision with material versatility, large area and high production rate. A large number of imprinted functional materials and related applications have been already described^5^,^6^. However, until now, due to the low mechanical stability and poor long-term durability, the exploitation of nanoimprinted functional structures in applications requiring long term or aggressive exposure has not been possible^7^. Moreover, despite their utilization potential, the enhancement of the mechanical properties of polymer nanoimprinted features has not received much attention and studies on mechanical properties such as hardness or abrasion resistance of artificial textures have been very limited^8^,^9^.

Here, we focus on the fabrication of nanoimprinted surfaces incorporating concurrently superhydrophobicity and self-cleaning (SH-SC) properties together with the enhancement in mechanical performance and electrical conductivity. There are numerous reports where SH-SC surfaces have been demonstrated based on different surface structural designs^3^,^5^. Additionally, there have been a few successful attempts to produce by scalable nanofabrication methods, mechanically resistant SH-SC surfaces. The most remarkable include strategies such as the implementation of micro size topographies^10^ or microbumpers for protection^11^ or the addition of fillers^12^ or lubricant coatings onto the surface textures^13^.

Our approach to prepare mechanically enhanced SH-SC surfaces is by texturing via thermal nanoimprinting nanocomposite materials based on different polymeric matrixes incorporating suitable nano fillers as reinforcing elements. We have chosen two different matrix-filler systems to investigate the grounds for the mechanical enhancement taken into consideration the imprint processing factors. The consumer plastics Polystyrene (PS) (amorphous) and polyvinylidene fluoride (PVDF) (semi crystalline), were chosen as matrix. A semicrystalline polymer was deliberately selected to investigate the influence of nanofillers on the degree of crystallinity of the polymer micro pillars that may account for a mechanical enhancement. The morphology of semicrytalline polymers regarding polymer chain or crystal orientation has seen to have an impact on the performance of semiconducting polymer on electronic or photovoltaic devices^14^,^15^.

Carbonaceous nanoparticles with different dimensionality were selected here as reinforcing fillers. 2D reduced graphene oxide (RGO) flakes for the case of PVDF and 1D single wall carbon nanotube (CNT) fillers for PS. On these two exemplary systems, we examine the influence of the nanoparticles on the flow induced crystallization during nanoimprint processing as well as the influence of the shear flow on the orientation of the CNT fillers as factors accountable for the mechanical enhancement of the imprinted pillars.

Besides enhancing the mechanical robustness, the fillers were chosen also to provide additional functionalities derived from their nature adding to the multi functionality of the material; in the present case of carbon materials, conductivity.

Merging the areas of nanocomposites and nanofabrication will allow for a variety of structural and functional properties combinations providing a new paradigm for multifunctional material design.

## Results and Discussion

RGO and CNTs, owing to their outstanding properties^16^, have shown to improve notably the mechanical properties of polymer composites^17^. Furthermore, due to the aspect ratio of the CNTs, they are ideal filler for the reinforcement of topographical features to transfer efficiently mechanical stresses to the bulk material. RGO was used as reinforcement filler for the semicrystalline PVDF. The mechanical reinforcement of different polymer matrices by graphene has been previously explored^18^,^19^. Because of the 2D morphology of the graphene platelets, mechanical enhancement due to direct stress transfer to the support film is not obvious. In this case, it is expected that the enhancement of mechanical properties takes place through changes in the semicrystalline polymer morphology (degree of crystallinity, spherulite morphology, crystallite size, etc.) due to presence of the filler^17^.

It is well known that the strong van der Waals interaction between nanoparticles favors the formation of aggregates, inducing a decrease in the efficiency of the reinforcement^20^. In order to obtain a good dispersion of the nanoparticles and avoid the formation of agglomerates, PS-CNT nanocomposites were prepared by *in situ* polymerization^21^. An improved dispersion has a direct impact on the polymer/nanotube interfacial interaction, which has shown to be a critical parameter for the stress transfer from the nanoparticles to the matrix^22^. In the case of RGO, it has been established that a well dispersed RGO renders improved properties in relation to strength and fracture toughness^23^.

**Figure 1:**
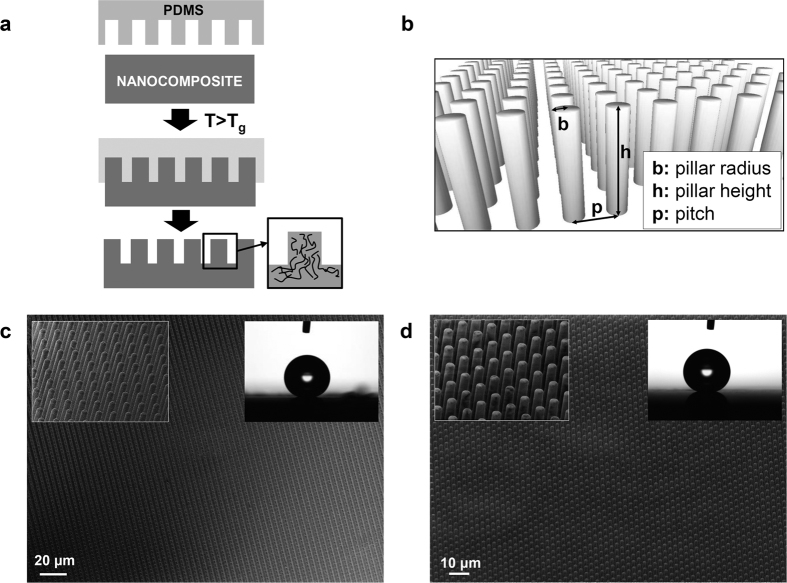
(**a**) Outline of the thermal nanoimprinting process to form surface structured nanocomposite films. (**b**) Illustration of pillar array indicating the dimensional parameters. (**c,d**) SEM pictures of the 2 μm pillars arrays imprinted on (**c**) PS and (**d**) PS-CNT films by thermal imprinting. The insets show close up images of the micropillars and images of 4 μl drops of water on the films surfaces exhibiting superhydrophobicity.

Figure 1a shows the outline of the nanoimprinting process employed to produce the SH-SC films. The geometrical parameters of the pillar features described in Fig. 1b, including pillar radius (*b*), height (*h*) and pitch (*p*) are summarized in Table 1. The parameters that influence surface wettability such as roughness and feature density are also included^24^. Equation (1) describes the roughness as the ratio of the actual surface area to its projected one. Assuming a regular array of long, dense cylindrical pillars it gives out a high value of 5.71


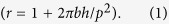


Feature density percentage is defined according to equation (2) as the ratio between the top and bottom surface areas


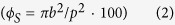


Figure 1c and d show scanning electron microscopy (SEM) images of the substrate surfaces obtained after imprinting the pure polymer (PS) and the nanocomposite respectively. In both cases, ordered arrays of well formed pillars are observed and no apparent roughness increase or defects on the surface of the composites pillars are appreciated. (*cf*. inset Fig. 1c,d), indicating that, during the imprinting process, the nanoparticles dispersed in the polymer matrix were carried by the polymer flow into the pillars.

### Wetting properties

The wetting properties of the imprinted films were characterized by water contact angle measurements. The static water contact angle (WCA), hysteresis and sliding angle observed for the pure matrix and their nanocomposites are listed in Table 2. As it can be observed, a superhydrophobic behavior is displayed by all substrates, with static water contact angles (WCA) >150°. In addition, for the PS-CNT composites, sliding angles <5° and hysteresis values <10° were observed and water droplets slid off the surfaces almost spontaneously. (See Supplementary video S1 and image S2). These values prove the strong superhydrophobic and self-cleaning character of these surfaces. Insets of Fig. 1c,d show images of drops of water deposited over the two surfaces.

### Dispersion characterization by confocal Raman spectroscopy

**Figure 2:**
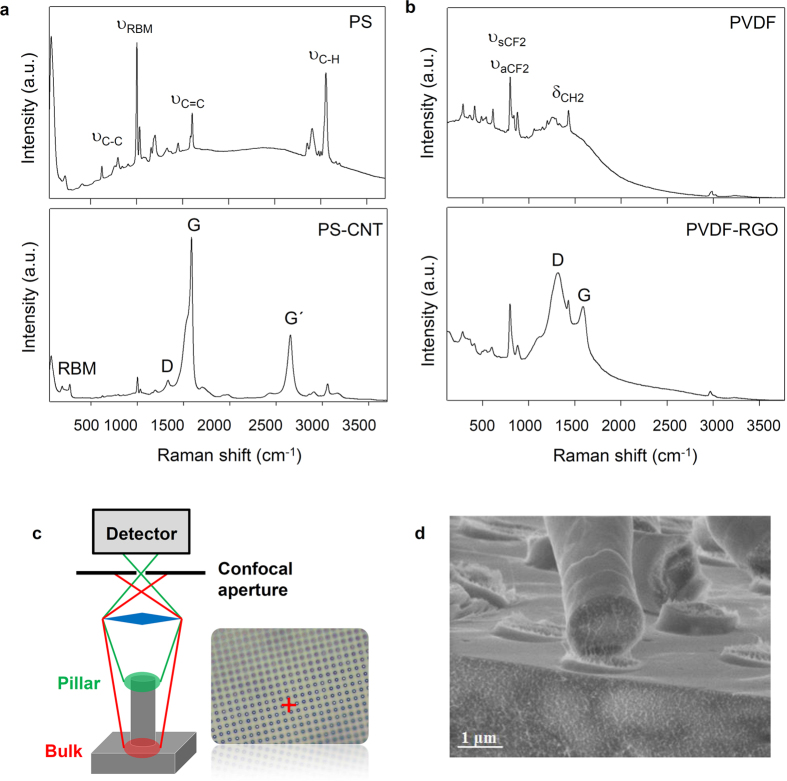
Raman spectra obtained from imprinted nanocomposites pillars of (**a**) PS and (**b**) PVDF and their composites (bottom part in each case). (**c**) Schematic view of confocal arrangement to obtain selectively spectral data from the pillars’ top at the focal point by blocking scattering light arising from bulk region. (**d**) SEM image of a fractured PS-CNT pillar substrate showing infiltrated carbon nanotubes into the pillars.

The dispersion of the nanofillers is a critical parameter for effective reinforcement, particularly in the case of a textured surface as it requires the filler to effectively enter the cavities of the mold with the polymer flow during the imprinting process. The distribution and incorporation of nanoparticles into the microtextured polymer film was studied by means of confocal Raman spectroscopy. This technique has proven previously to be useful for the study of the dispersion of nanotubes in 1D (nanorods) confined nanocomposites^25^. Figure 2 shows the confocal Raman spectra corresponding to the pure matrix (PS and PVDF) and their nanocomposites (top and bottom respectively). The main bands are labeled. For micropillars reinforced with nanotubes, the characteristic bands of SWCNT, namely G, G′, D and radial breathing modes (RBM)^26^,^27^ are clearly observed, confirming the presence of nanoparticles inside the micropillars. The constant signal of CNT in the different areas measured indicates a good dispersion of the filler into the polymer matrix. Similar results are observed for the dispersion of the RGO flakes into the PVDF micropillars. It can be seen that the characteristic signals (D, G)^28^ of the graphene sheets coexist with the PVDF bands (Fig. 2b).

The depth resolution of confocal Raman microscope can be calculated according to the equation (3)^29^


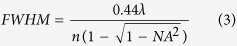


where FWHM is the full width at half maximum of the confocal depth profile, λ is the laser wavelength, n the refraction index and NA the numerical aperture of the microscope objective. Hence, with a laser wavelength of 532 nm the depth resolution achieved is *ca.* 400 nm which allowed us to detect the nanoparticles inside the pillars. As illustration, a schematic view of the confocal principle is shown in Fig. 2c, whereby adjusting the focal point on the top of the pillars, the spectral data was obtained only from nanotubes inside the micropillar.

In order to observe the filler dispersion within the reinforced pillars, a simple fracture was produced on the imprinted PS-CNT substrate and SEM images of the cross-section were taken. One of the images displayed in Fig. 2d, shows the dispersion of CNTs throughout the polymer matrix as well as their inclusion into the pillars. It is worth remarking that, after the fracture procedure, many of the broken pillars remained attached to the substrate on their original positions where nanotubes connecting the body of broken pillars to their bases can be appreciated. The orientation of the visible CNTs was parallel to the main axis of the micropillars, indicating the presence of strong shear forces during the filling of the mold cavities. Indeed, shear has been proven to be a very effective method for the orientation of nanotubes in melt processed polymers^30^. This longitudinal orientation adopted by the nanotubes along the pillar axis is optimal to transfer mechanical stresses from the pillar to the bulk film. The mechanical enhancement attained is substantiated in the next section by mechanical measurements.

### Mechanical characterization

To study the reinforcement effect by the nanofillers on the imprinted textures, their mechanical properties were evaluated by performing nanoindentation and nanoscratch tests by means of a nanoindentation tool. These techniques have been employed previously to evaluate the mechanical properties of polymer composites^31^,^32^,^33^ and have been employed in the mechanical characterization of random micro and nano textured polymeric surfaces produced by plasma etching^9^,^34^,^35^.

Typical nanoindentation tests involve the continuous measurement of force and depth of penetration as a probe is driven into the sample surface during loading and unloading^36^. In this work, a cono-spherical diamond tip with a radius of 10 μm and a self-included angle of 60° was employed as mechanical probe. In order to determine the appropriate maximum load for the PS and PVDF pillars without interference from the underlying substrate, the minimum critical buckling load was calculated for the PS and PVDF pillars, assuming uniaxial deformation. The minimum critical buckling load (*F*_*c*_) for Mode I buckling of a cylindrical pillar is defined by equation 4


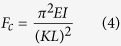


where E is the Young’s modulus (3.2 and 1.5 GPa for PS and PVDF respectively) and the area moment of inertia of the cross section of the pillar, I, is defined by equation (5)


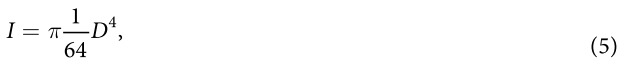


being L and D the length and diameter of the pillar, respectively, and K the column effective length factor, whose value depends on the conditions of end support of the column (K = 1 for both ends immobilized). From this, the calculated buckling loads for PS and PVDF pillars were 172 and 81 μN respectively. As such, the maximum load used for nanoindentation tests was limited to 200 μN in order to identify the onset of buckling and the departure from the uniform elastic response of the pillars, so that the stiffness and elastic modulus were determined from the initial elastic linear response prior to buckling. Due to the complex response that can be obtained from textured surfaces and the uncertainty in the number of pillars in contact with the mechanical probe, the comparison of the mechanical properties between the pure matrix and the reinforced nanocomposite was done on the basis of the substrate “stiffness” defined as the rate of change of depth with load during elastic regime of the nanoindentation. Nanoscratch tests were also performed by dragging the indenter probe laterally while an increasing normal load was applied. The normal displacements (scratch depth) as well as the normal and lateral force signals were continuously acquired during the tests. This data set allows for testing properties such as scratch resistance and critical load for micropattern failure, which can be typically identified as a discontinuity in the lateral force and referred to as the critical load of failure. Figure S3 shows the force profile used during the measurements.

**Figure 3:**
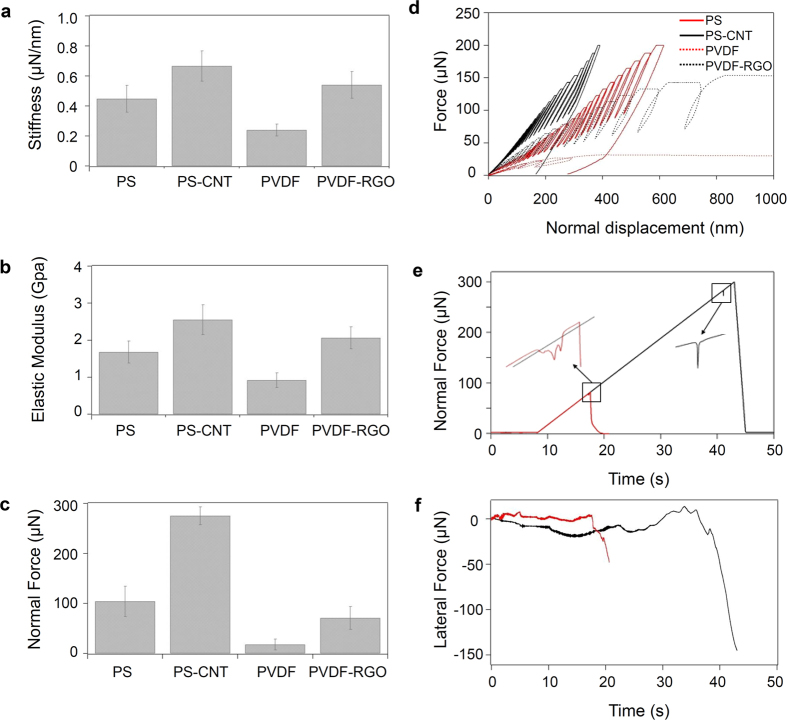
(**a**) Contact stiffness, (**b**) elastic modulus (Ep) and (**c**) critical failure load (CFL) measured for PS, PVDF and their composites. The values in (**a**) and (**b**) were obtained from the analysis of the low penetration region of the nanoindentation curves, within the initial linear elastic region. Values in (**c**) were obtained from nanoscratch tests with ramping normal force. (**d**) Representative nanoindentation loading-unloading curves obtained for PS, PVDF and their microstructured composite surfaces, showing the initial elastic linear region and the onset of buckling of the micropillars. (**e**) Representative normal load ramping for nanoscratches made in PS and PS-CNT. Insets show in detail the presence of bumps prior to the sharp decrease in normal force that are identified as signature of failure to determine the critical failure load. (**f**) Lateral force signal obtained during the scratches shown in (**e**).

Figure 3 summarizes the mechanical characterization data obtained from the different substrates. Figure 3a,b shows the contact stiffness (S) and elastic modulus (E_p_) for the imprinted surfaces. Figure 3c shows the normal force measured at the pillar failure load during nanoscratch tests performed on the different substrates. Representative curves of the load-unload nanoindentation cycles performed on the different substrates, are shown in Fig. 3d. The figure shows that the initial load-depth response was linear elastic up to a critical load; this load marks the buckling of the pillars in each case. Hereafter, the surface stiffness could be determined in each case from the slope of the initial loading segment. It is apparent from the graph that the surface stiffness of the nanocomposites, that is, the applied load for the same penetration depth, was much lower than that of the pristine polymer matrix, which is an evidence of the reinforcement effect of the nanoparticles, considering that the geometrical parameters of the micropillars were identical in all cases.

Estimation of the elastic modulus in each case required to elucidate the number of pillars in contact. At typical indentation depths within the initial elastic contact regime, of the order of 60 nm, and considering the geometry of the sphero-conical probe with tip radius of 10 μm, the total contact area results in *ca*. 3.8 μm^2^. Considering the diameter and pitch of the microstructured surface (Table 1), it is safe to assume that at initial contact, only one of the pillar established contact with the probe.

The elastic modulus, Ep, was then calculated from the stiffness of a cyclindrical pillar, according to equation (6)


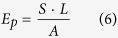


where S is the stiffness, A is the cross-sectional area of the pillar, and L is the pillar length.

The values obtained indicate a noticeable reinforcement effect of the nanoparticles in both cases. Figure 3a and b show a considerable increase in stiffness and Young’s modulus for both composites with 50 and 100% improvement with respect to the pristine polymers for the PS-CNT and the PVDF-RGO composites respectively.

Figure 3c shows the critical loads for failure estimated from the nanoscratch tests. Figure 3e,f plots the variation of the prescribed normal force and the resulting lateral force for two of the scratches. For clarity, only one curve corresponding to the PS (red) and one for the PS-CNT (black) micropillar substrates are presented. In each case, the normal load was ramped during the scratch until failure of the microstructure was detected (*cf*. Fig. 3e), whose signature was a bump in the normal load (*cf.* insets Fig. 3e), designated as critical failure load (CFL), and a dramatic drop in the lateral force sensed by the mechanical probe (*cf.* Fig. 3f). The CFL is here associated with the failure (fracture or plastic deformation) of one or several micropillars during the nanoscratch (An image of such failure is shown in Figure S4). Figure 3c clearly demonstrates that the CFL is much larger for the reinforced micropillars than for the pristine polymer ones, indicating that the presence of fillers in the imprinted pillars causes a significant increase of the force necessary for microstructure failure.

### Crystalinity characterization

As discussed above, the improvement in the mechanical properties observed for the PVDF-RGO composite was particularly remarkable. Previous works have ascribed the reinforcement of RGO on PVDF composites to the formation of an ordered polymer interfacial structure by the RGO nanosheets^37^. In addition, an enhanced crystallization of PVDF induced by the RGO can be expected. Indeed, it is well recognized that nanoparticles are effective nucleating agents for various semicrystalline polymers influencing both, the crystallization kinetics and crystalline morphology^38^. And particularly for PVDF, there are reports demonstrating that the addition of graphene nanosheets can nucleate its crystallization^39^,^40^. The impact of the crystalline morphology on the mechanical properties of polymer composites is well documented and generally the nucleation of polymer crystals leads to an improved interfacial shear strength between the nanoparticles and the polymer which results in a superior reinforcement^22^,^41^. In adittion, the crystalline phase tends to increase stiffness and tensile strength values^42^. In order to elucidate the degree of crystallization for the imprinted PVDF-RGO pillars, grazing incidence wide angle x-ray scattering (GIWAXS) was employed, using synchrotron radiation.

**Figure 4:**
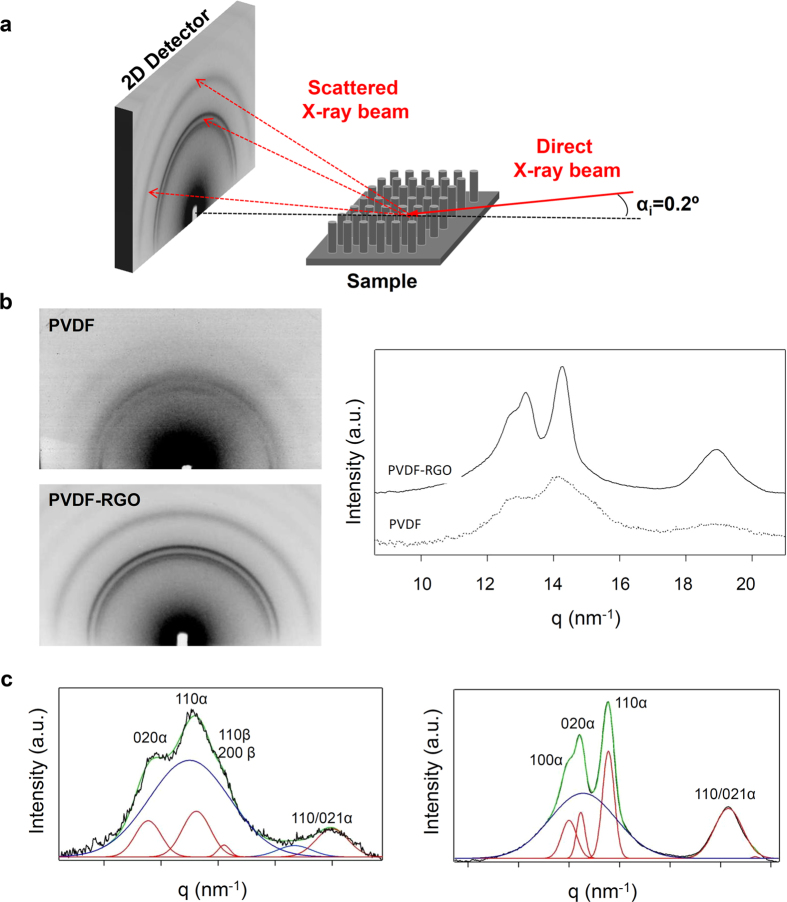
Characterization of the degree of crystallization for PVDF-RGO imprinted surfaces. (**a**) Schematic view of the GIWAXS measurement. The small grazing incidence angle used (α_i_ = 0.2°) allow to obtain information from the substrate topography. (**b**) 2D diffraction patterns of PVDF and PVDF-RGO (left) and their corresponding integrated scattered intensity as a function of the scattering vector (right). (**c**) The deconvolution of the 1D integrated intensity curves. Peak deconvolution and indexing was performed assuming the existence of two different crystalline phases for the pure PVDF matrix in agreement with the Raman characterization.

The grazing incidence geometry has been widely used for the characterization of polymer surfaces and interfaces properties, including surface crystallinity of polymer films^43^,^44^. The grazing geometry ensures that the x-ray beam impinges onto the substrate surface with a very low incident angle, allowing to extract information from the substrate surface, in this case that is, from the pillar topography (see illustration in Fig. 4a).

Figure 4b shows the integrated scattered intensity as a function of the scattering vector q (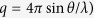 obtained from the azimuthal integration of the 2D diffraction patterns for the PVDF and its composite with 1% w/w of RGO (Fig. 4b-left). The degree of crystallinity has been calculated according to the equation (7)


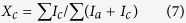


where I_c_ is the integrated area underneath the crystalline peaks and I_a_ is the integrated area of the amorphous halo, estimated from the peak convolution used to fit the intensity profiles, as shown in Fig. 4c. The peak indexing was done based on previous works on PVDF nanocomposites^45^. The measured d-spacings observed for the nanocomposite are coincident with those expected for the unit cell corresponding to the pure α phase of the PVDF (*cf*. Table S5). In the case of the pure polymer matrix, the peak indexing was performed assuming the existence of two different crystalline phases, α and β (*cf*. Table S6). The presence of the piezoelectric polymorph of the PVDF was confirmed by means of Raman spectroscopy (see Figure S7), which allow us to detect the formation of the β phase by the appearance of a characteristic strong band located at 839 cm^−1^.

While the pure matrix shows the coexistence of α and β phases, the crystallization of the β ferroelectric phase seems to be hindered by the presence of RGO nanparticles under the processing conditions. The degree of crystallization of the pristine PVDF within the pillars was then estimated to be less than 25%, similar to that observed in PVDF crystallized under confinement^46^, while the degree of crystallization for the nanocomposite was calculated to be *ca*. 45%.

This higher degree of crystallization for the PVDF-RGO composite induced by the filler nucleating effect^47^ explains the increased mechanical parameters values measured compared to those of the pristine PVDF. It could also be expected that due to the shear flow of the polymer during filling, the polymer chains would adopt an orientation along the pillar axis, giving rise to a guided crystallization upon solidification^46^. However, as seen in Figure 4b, the isotropic distribution of the scattered intensity along the azimuthal angular range indicates that there was no preferential orientation of the PVDF crystals inside the micropillars.

In our particular case of process conditions, the confinement imposed by the micron size pores is deemed insufficient and the chain relaxation is likely to take place due to the high processing temperature and slow cooling rate prior to demolding.

### Electrical characterization

The conducting properties of both nanocomposites were evaluated by broadband dielectric spectroscopy^47^,^48^. The characterization results showed that for the PVDF-RGO composite, the conductivity follows a linear dependence with frequency with a slope close to one (see Supplementary Figure S8). This behavior is characteristic of insulating materials, therefore we can assume that the concentration of RGO employed was too low to establish a connected network of RGO nanoplatelets. However, the imprinted PS-CNT composite showed a frequency independent AC-conductivity behavior below certain frequency (see Figure S4), confirming the superior efficiency of CNT to form a conducting percolation network. The conductivity values obtained for the PS-CNT nanocomposite at the lower frequency measured, which is equivalent to that obtained under DC conditions, was *ca*. 10^−4^ S · cm^−1^. This conductivity is adequate to confer antistatic properties to the material.

## Conclusion

In summary, we have demonstrated the fabrication of multifunctional superhydrophobic and self-cleaning surfaces with enhanced mechanical and electrical performance based on surface nanoengineering of high aspect ratio surface structures on CNT and RGO reinforced nanocomposites.

Raman and SEM studies confirmed the good dispersion of the fillers within the polymer matrices and the presence of fillers inside the surface structures. GIWAXS measurements on the PVDF-RGO micropillars confirmed an increased degree of crystallinity over the pristine PVDF underpinning the mechanical enhancement.

The integration of suitable nano fillers and nanocomposite formulations into nanoengineered surfaces with function design topographies may enable the production of multifunctional surfaces with adequate mechanical durability for industrial acceptance and product manufacture in applications ranging from optoelectronics, building materials to the biomedical field.

## Methods

### Fabrication

The polystyrene nanocomposite contained 0.35% in weight of Single Wall Carbon Nanotubes synthesized by using the HiPco method (CNI Technology Co,TX, USA). The composite was obtained by *in situ* radical polymerization process using azobisisobutyronitrile as initiator according to previous procedures. After oxygen was removed by freeze-thaw cycles, the reaction flask was placed into an oil bath preheated at 60 °C. Reaction of styrene polymerization was accomplished under inert atmosphere during 20 hours with constant stirring. The composites were coagulated by the drop wise addition of the solutions into a large volume of methanol (MeOH) while intense stirring. The composite precipitated immediately due to the poor polystyrene solubility in MeOH and the nanotubes get entrapped by the polymer chains in the process. The composite was then filtered, washed repeatedly with MeOH and dried in vacuum overnight.

The polyvinylidene fluoride (PVDF, Kynar-Arkema) nanocomposite with 1% in weight of reduced graphene oxide (RGO, Graphenano, mean particle size 1–3 μm) was prepared by the coagulation method. Initially, a certain amount of RGO powder was dispersed in dimethyl acetamide (Scharlau) using a sonication bath. Based on the desired weight fraction of the filler in the composite, a certain volume of the dispersion was added to solutions of PVDF prepared in the same solvent. The polymer solution including the RGO dispersion was stirred using Ultraturrax tool (10.000 rpm, 10 minutes) and ultrasonic bath (10 minutes), repeating the cycle 3 times to ensure a good dispersion. The nanocomposite was coagulated by dropwise addition of the solution into a large volume of distilled water (5:1, water:DMF) while intense stirring was performed. The precipitate was filtered using Buchner funnel, washed with H_2_O and dried on a hot plate at 70 °C for 3 hours. The residual solvent was removed by vacuum drying at 80 °C for 6–8 hours.

Films (*ca*. 200 μm thick) of pure matrix and their composites were prepared by melt pressing using a heating stage. Thinner films were prepared over Si wafers by spin coating from CHCl_3_ solutions (50 mg · ml^−1^) with thicknesses ranging from 200 nm up to 5 μm. The microstructured substrates were prepared by thermal nanoimprinting using PDMS replica molds (obtained from a Silicon master mold) with pore size of 2 μm diameter and 12 μm length. The nanoimprinting processes were accomplished by using an EITRE Nano Imprint Lithography system (Obducat). The thermal imprinting conditions were T = 130 °C; P = 30 bar and t = 5 min for polystyrene and T = 140 °C; P = 30 bar; t = 5 min for its nanocomposite with CNT. The microstructured films of PVDF-RGO composites were prepared by thermal imprinting using a CNI nanoimprint tool (NILT) at T = 180 °C; P = 9 bar; t = 15 min. After the imprinting process, the temperature was decreased down to 70 °C prior to demolding at room temperature.

### Wetting characterization

The surface morphology and detailed structural features of the imprinted micropillars were characterized by scanning electron microscopy (EVO MA15, Zeiss). The static and dynamic contact angle measurements were performed using an optical tensiometer (Attension Theta, Biolin Scientific). Deionized water droplets of 4 μL were gently deposited onto the substrate to prevent the drops from rolling off. Advancing and receding WCA measurements were performed by using the tilt base method using a stage tilting speed of 90°/min. The static, advancing and receding angles were measured by adjusting the drop profile to a Young-Laplace curve. The contact angle hysteresis was calculated as the difference between the advancing and receding contact angles measured just before drops rolled off.

### Optical characterization

A confocal Raman microscopy (SENTERRA, Bruker) was employed to study the dispersion and location of carbon nanotubes on the microstructured nanocomposite films at wavelengths: 532 and 785 nm.

The GIWAXS measurements on the microstructured substrates of PVDF and PVDF-RGO (1% w/w) were performed at the BM26 beamline of the European Synchrotron Radiation Facility (ESRF). An x-ray beam a wavelength of 1.03 Å was used. 2D X-ray patterns were collected with a CCD detector (FReLoN). The norm of the q reciprocal-space vector was calibrated using Al_2_O_3_ as a standard reference. An incidence angle of 0.2°, close to the value of critical angle of polymers, was used to study the substrate to ensure that the structural information is collected from the pillar array.

### Mechanical characterization

The mechanical properties of the pillars were evaluated by performing nanoindentation and nanoscratch tests using a Hysitron TI-950 TriboIndenter. All the measurements were carried out with a spherical diamond probe with tip radius of 10 μm. Each indentation test consisted of 20 load-hold-unload cycles with times of 1–1–1 s respectively, with an unloading to 50% of maximum load for each cycle up to a maximum final load of 200 μN. A total of 15 indentations were carried out per substrate. Nanoscratch tests were performed by moving the nanoindentation probe laterally while applying a ramping variable normal load at a constant loading rate. Initially, the probe performs a pre-scan test at a low load of 2 μN, which is later used to correct the sample tilt. Scratches of 12 μm in length were performed while varying the normal applied load from 0 up to 300 μN. 12 measurements were performed on different areas. The maximum load was chosen according to the penetration depth values measured during the nanoindentation tests to ensure a tip penetration of at least 10% of the pillars’ height, i.e 1.2 μm.

### Electrical characterization

Circular gold electrodes (10 mm or 20 mm in diameter) were deposited by sputtering the metal onto both free surfaces of PS-CNT and PVDF-RGO films (650 μm thick). The complex permittivity ε* = ε′ − iε″, where ε′ represents the permittivity and ε″ the dielectric loss, was measured as a function of frequency (10^−1^ Hz < F < 10^6^ Hz, being F the frequency of the applied electric field) at room temperature by using a Novocontrol broadband dielectric spectrometer. Electrical conductivity, σ, was derived by σ(F) = ε_0_2πFε″ where ε_0_ is the vacuum permittivity.

## Additional Information

**How to cite this article:** Hernández, J. J. *et al*. Multifunctional Nano-engineered Polymer Surfaces with Enhanced Mechanical Resistance and Superhydrophobicity. *Sci. Rep.* **7**, 43450; doi: 10.1038/srep43450 (2017).

**Publisher's note:** Springer Nature remains neutral with regard to jurisdictional claims in published maps and institutional affiliations.

## Supplementary Material

Supplementary Information

Supplementary Video 1

## Figures and Tables

**Table 1:** Geometrical parameters of the imprinted pillars.

Diameter [μm]	Height [μm]	Pitch [μm]	Roughness	Density
2	12	4	5.71	20%

**Table 2:** Wetting properties of PS and PS-CNT micro pillared surfaces.

Substrate	WCA°	Sliding angle°	Hysteresis°
PS	172 ± 2	6 ± 1	5 ± 2
PS-CNT	173 ± 1	3 ± 2	6 ± 2
PVDF	155 ± 2	18 ± 2	19 ± 6
PVDF-RGO	157 ± 1	12 ± 3	11 ± 4
