# Supplementary material for: Multifunctional Nano-engineered Polymer Surfaces with Enhanced Mechanical Resistance and Superhydrophobicity
Source: Sci Rep. 2017 Mar 6;7:43450. doi: 10.1038/srep43450 (PMC5337973; doi:10.1038/srep43450)
Supplement: Supplementary Information [file srep43450-s1.pdf]

Supplementary Information

Multifunctional Nanoengineered Surfaces with Enhanced Mechanical Durability and Superhydrophobicity

Jaime J. Hernández[1], Miguel A. Monclús[2], Ivan Navarro-Baena[1], Felipe Viela[1], Jon M. Molina-Aldareguia[2], Isabel Rodríguez[1]*

[1]Madrid Institute for Advanced Studies in Nanoscience (IMDEA Nanoscience), C/Faraday 9, Ciudad Universitaria de Cantoblanco. 28049 Madrid
[2]Madrid Institute for Advanced Studies in Materials (IMDEA Materials), C/ Eric Kandel, 2, Tecnogetafe, Getafe. 28906 Madrid

Supplementary Video S1 Video of a water droplet sliding off a PS-CNT high aspect ratio micropillared surface

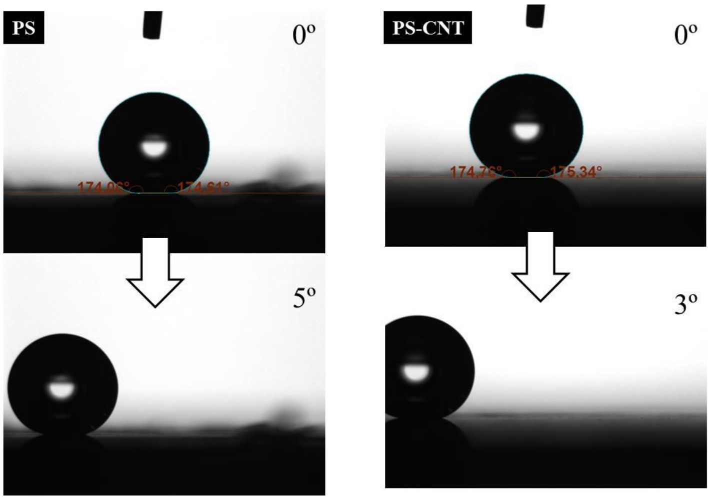

Supplementary Figure S2. Images of 4 µL droplets sliding over PS and PS-CNT composite imprinted micro pillar surfaces. Tilting angles are indicated in each image.

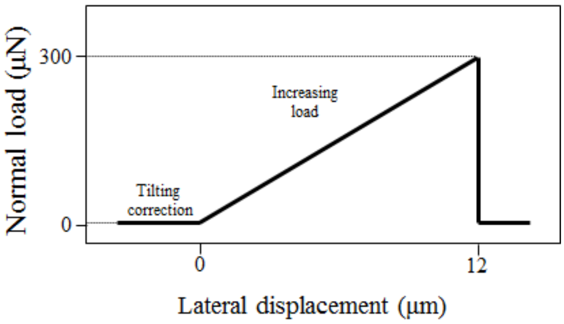

Supplementary Figure S3. Load profile employed during nanoscratch tests.

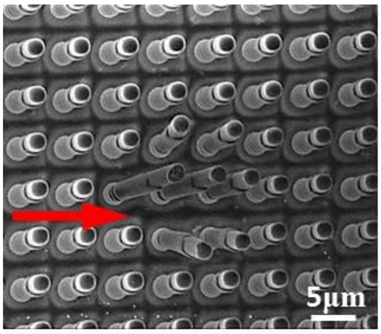

Supplementary Figure S4 SEM image of the indentation left during the nanoscratch tests performed in a PS-CNT microstructured film. Red arrow indicated the scratch direction.

Supplementary Table S5. Structural parameters of the PVDF-RGO nanocomposite derived from the X-ray measurements

**hkl**	**d_{exp} [nm]**	**d_{calc} [nm]**
**100 α**	0.49	0.50
**020 α**	0.47	0.48
**110 α**	0.44	0.44
**120/021 α**	0.32	0.32

Supplementary Table S6. Structural parameters of the PVDF derived from the X-ray measurements

**hkl**	**d_{exp} [nm]**	**d_{calc} [nm]**
**100α**	0.49	0.50
**10 α**	0.44	0.44
**110/200 β**	0.43	0.43
**120/021 α**	0.32	0.32

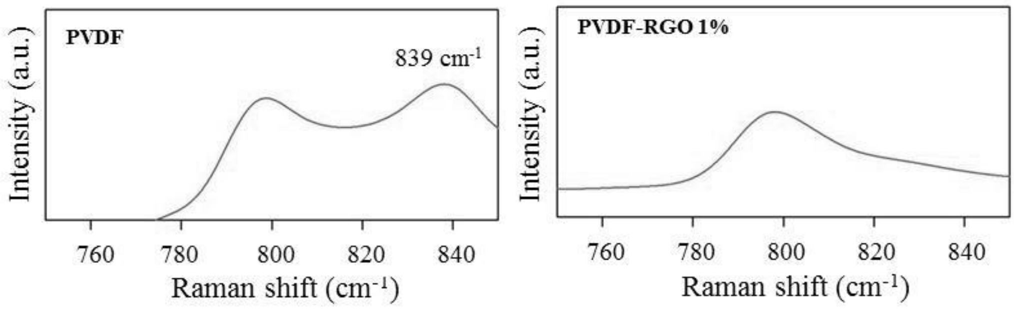

Supplementary Figure S7. Enlarged region of Raman spectra of PVDF and its RGO composite. The band located at 839 cm^{-1}, which is strong on the β-phase,[1] is only observed in the pure PVDF matrix.

[1] Boccaccio, T.; Bottino, A.; Capannelli, G.; Piaggio, P. *Journal of Membrane Science* **2002,** 210, (2), 315-329.

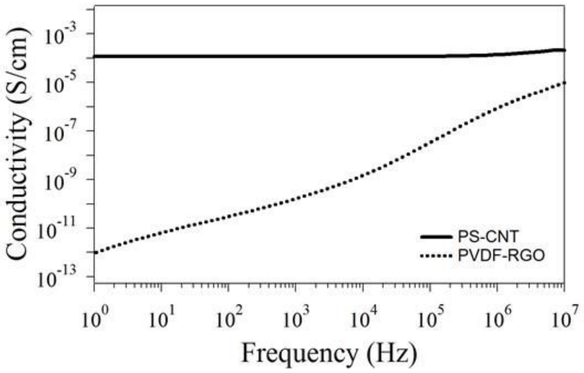

Supplementary Figure S8 Broad band electrical conductivity, σ(F) as a function of frequency, F, for nanocomposites of PVDF-GNP and PS-CNT

Calculation of the buckling load according to Equation S1:

$$F = \frac{\pi^2 EI}{(KL)^2} \qquad \text{(S1)}$$

F = maximum or critical force (vertical load on column),
E = modulus of elasticity,
I = area moment of inertia of the cross section of the rod,
L = unsupported length of pillar
K = column effective length factor, whose value depends on the conditions of end support of the column. For both ends pinned (hinged, free to rotate), K = 1.0.
D= pillar diameter
KL is the effective length of the column.

E_{PS}=3.2 GPa
E_{PVDF}=1.5 GPa

$$I = \pi \frac{1}{64} \pi D^4$$

$$F_{PS} = \frac{\pi^2 3200 \, {}^N/_{mm^2} \, 2\pi \frac{1}{64}\pi(0.002)^4 \, mm^4}{(1\cdot 0.012)^2 \, mm^2} = \mathbf{172\mu N}$$

$$F_{PVDF} = \frac{\pi^2 1500 \, {}^N/_{mm^2} \, 2\pi \frac{1}{64}\pi(0.002)^4 \, mm^4}{(1\cdot 0.012)^2 \, mm^2} = \mathbf{81\mu N}$$
